# Formaldehyde and its surrogates as a C1 platform for defossilised modern societies

**DOI:** 10.1039/d5cs00882d

**Published:** 2025-11-10

**Authors:** Andrea Rodil, Jan Deska, Martin H. G. Prechtl

**Affiliations:** a Department of Chemistry, University of Helsinki, Chemicum A. I. Virtasen aukio 1 00560 Helsinki Finland jan.deska@helsinki.fi https://www.deskalab.com; b Centro de Química Estrutural (CQE) and Centro de Recursos Naturais e Ambiente (CERENA), Instituto Superior Técnico, Universidade de Lisboa Av. Rovisco Pais 1 1049-001 Lisboa Portugal martin.prechtl@tecnico.ulisboa.pt https://tecnico.ulisboa.pt https://www.h2.bio; c Instituto Superior de Engenharia de Lisboa, Instituto Politécnico de Lisboa 1959-007 Lisboa Portugal; d Albert Hofmann Institute for Physiochemical Sustainability, Akademie zur Förderung Physiochemischer Nachhaltigkeit e.V. Albert-Schweitzer-Str. 22 32602 Vlotho Germany https://www.a-h.institute

## Abstract

This tutorial serves as an accessible introduction for researchers and students interested in the multifaceted chemistry of formaldehyde and its potential in shaping a more sustainable future. We explore its roles in renewable energy storage in the form of liquid organic hydrogen carriers (LOHCs) and renewable fuels, as well as carbon capture, utilization and storage (CCUS), and biomass valorisation. Furthermore, the relevance of these applications to several United Nations Sustainable Development Goals (UNSDGs 6, 7, 9, 12, and 13) is examined. Beyond the energy and environmental aspects, we discuss the use of formaldehyde and related surrogates in synthetic chemistry, focusing on innovative catalytic strategies to make use of this versatile and abundant C1 building block. Given formaldehyde's central role as an intermediate in both synthetic and biological C1-H_2_ reaction networks, the tutorial additionally offers discussion points on related small molecules, including methane, methanol, formic acid, CO, and CO_2_.

## Abstract 2

This tutorial serves as an accessible introduction for researchers and students interested in the multifaceted chemistry of formaldehyde and its potential in shaping a more sustainable future.

## Graphical abstract

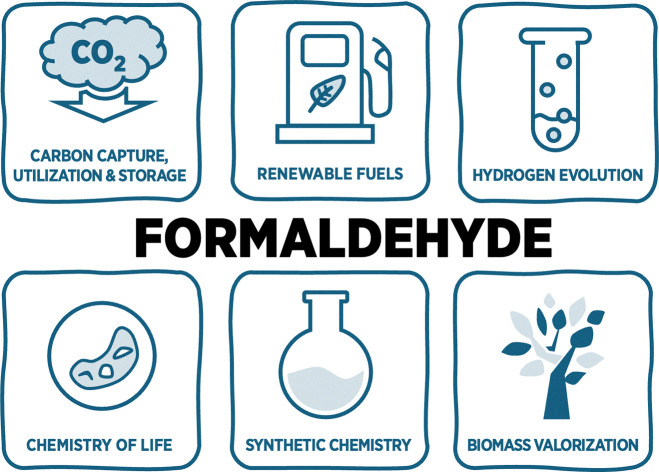

**Key learning points**

1. Biological and anthropogenic sources of formaldehyde and their relevance in nature and for modern societies.

2. Formaldehyde and its surrogates – production and utilisation in industries.

3. The formaldehyde toolbox: opportunities in R&D for synthetic transformations.

4. Current developments and opportunities for hydrogen storage with C1-molecules coupled with CCUS.

5. Future opportunities and challenges for sustainable chemistry and the potential for the energy transition.

## Introduction

**Scheme 1:**
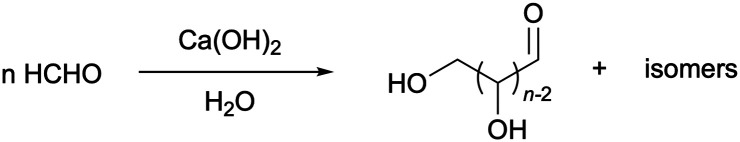
Formose reaction, discovered by Butlerov (1861)^14^ and reaction pathways further clarified by Breslow (1959).^12^

Formaldehyde (H_2_CO) is the simplest aldehyde and one of the most abundant molecules in the universe, where it is believed to play a crucial role in the origin of life on this planet.^1–6^ Its formation traces back to 13.7 billion years ago when it was among the first molecules to exist in a universe under abiotic conditions.^1^ Formaldehyde, albeit not bioaccumulative, acts as a reactive intermediate for the formation of various biologically relevant molecules such as carbohydrates and amino acids, thus serving as a key molecule in the development of nucleobases and other complex biomolecules like ribose and other sugars.^2,4,6,7^ In the interface between the living and the inanimate world where abiotic reaction pathways in the presence of inorganic bases or minerals lead to complex organic matter,^8–11^ the Formose reaction is a general pathway to convert formaldehyde into higher carbohydrates (Scheme 1).^2,4,6,7,10,12–20^

Formaldehyde also represents an intermediate in the pathways of biological carbon fixation, CO_2_ reduction, through photosynthesis as part of the Calvin–Benson cycle,^21–24^ with ribulose-1,5-bisphosphate carboxylase/oxygenases (RuBisCO) as central enzymes for the CO_2_ conversion and O_2_ evolution.^25^ Other sources of formaldehyde originate from the decomposition of biological and anthropogenic organic matter,^26–29^ where the emission of formaldehyde to the atmosphere, soil and water takes us to the more problematic aspects of our lead actor in this tutorial.

In an aqueous environment, formaldehyde exists almost exclusively in its hydrated form methanediol (H_2_C(OH)_2_) (>99%), along with some higher oligomers and only trace amounts of free non-hydrated formaldehyde.^27,29,30^

As such formaldehyde hydrate is involved in biological energy conversion processes, such as methylotropic cycles wherein it acts both as a hydride source for NAD(P)H generation and as C1-entity for the formation of carbohydrates.^31^ Moreover, formaldehyde is a key intermediate in biological detoxification processes.^32,33^ In the aforementioned pathways for decomposition, energy conversion and detoxification, the terminal product is carbon dioxide and formaldehyde is therefore not bioaccumulative. These processes are essential for the life of mammals and other species. Albeit formaldehyde is often only mentioned with regard to its toxicity, actually, a human being has an estimated natural turnover of formaldehyde of 878–1310 mg kg^−1^ bodyweight per day with an estimated half-life of approx. 1–1.5 min. Here, only a fraction of <2 mg kg^−1^ is related to the intake through daily nutrition,^34^ underlining its central role as a transitory puzzle piece in both anabolic and catabolic processes in not just the human body, but across all kingdoms of life.^32,35,36^ At the same time, one needs to take into account the early conclusion by Paracelsus that “the dose makes the poison”, a fact also true for formaldehyde. Health-related concerns of anthropogenic formaldehyde emissions impact not only the wellbeing of employees in formaldehyde industries, but more generally the broader population and end-users.^29,37–43^ This relates, not only to emissions of gaseous formaldehyde to the atmosphere, but also to water contamination, with the necessity for the removal of formaldehyde from wastewater streams down to a very low, safe level of <40 ppm.^27,28,30,44–49^

Taking the above considerations into account, the natural energy conversion and detoxification pathways for formaldehyde formation and decomposition may serve as inspiration for artificial procedures in industries and R&D activities to create processes that do not consume large amounts of energy and eliminate toxic substances from the waste streams and supply chains. While it seems unlikely that we would see major efforts to intervene in natural processes related to formaldehyde emissions, one can certainly improve the anthropogenic formaldehyde production processes, utilisation and emissions in industries and products in a multi-billion dollar market which surpassed >30 megatons annually, and a forecast for an annual growth of 4%, to enable the production of various formaldehyde based-resins (>75%) and other bulk chemicals established and used in modern societies.^27,50,51^ Notably, more than 35% of the global annual production of methanol is used in order to fulfil the formaldehyde global demands. In the 1880s, formaldehyde was initially used as a medical preservative and has since been established for the production of synthetic materials, such as Bakelite (1907, phenol-formaldehyde resin invented by the Belgian chemist Baekeland). Now, formaldehyde is a crucial building block and cross-linker in >50 industrial processes, for the production of daily life commodities.^27^ This includes applications in the healthcare (germicides/disinfectants) and pharmaceutical areas, but also for the production of other chemicals relevant, for many sectors such as paints, inks, cosmetics, resins, and polymers/adhesives, besides many others for the production of furniture, construction, automotives, airplanes and textiles, to cite a few.^52–54^ In this context, one can distinguish between the requirements in some industries, where formaldehyde is unavoidable for the production of certain substances, and the urgent need to defossilise industry. A balance might be achieved by considering greener chemicals, or even maybe, new applications for the energy transition, energy storage (e-fuels, hydrogen storage) and the use of renewable sources whenever possible.

## Limitations of reaction conditions and mitigations

Three major application pathways can be identified when it comes to embracing formaldehyde's potential new roles and opportunities for energy storage and as a versatile synthetic intermediate: (i) enzymatically catalysed formation of formaldehyde during carbon fixation, (ii) its release during biomass fractionation and decomposition, (iii) formation catalysed by inorganic species under ambient, thermal or photo-induced conditions. These pathways need to take into account the properties, reactivity and stability (*vide infra*) of formaldehyde and its surrogates in comparison to other C1-molecules, namely methanol and formic acid, which can be also produced from renewables such as carbon dioxide directly or indirectly.^51,53,55–58^

### Reaction temperature and pressure

The relatively high reactivity of formaldehyde in comparison to methanol is interesting for both synthesis and low-temperature energy conversion processes, allowing for mild reaction conditions. For example, the dehydrogenation of methanol to carbon dioxide is endergonic while the dehydrogenation of formaldehyde and formic acid is exergonic (Table 1;^59^ for more discussion refer to the section ‘hydrogen evolution and energy storage’). Moreover, low temperature conversion processes can be advantageous over for example high temperature methanol dehydrogenation where the intermediate formaldehyde may suffer terminal decarbonylation rather than dehydrogenative decarboxylation (Table 1).

**Table 1:** Comparison of thermodynamic data^59^

Reaction	Δ*H*_r_ [kJ mol^−1^]	Δ*G*^0^ [kJ mol^−1^]
CH_3_OH + H_2_O → CO_2_ + 3	38.8	8.8
H_2_CO + H_2_O → CO_2_ + 2H_2_	−35.8	−47.4
HCO_2_H → CO_2_ + H_2_	−14.9	−31.8
H_2_CO + H_2_O → H_2_C(OH)_2_ → HCO_2_H + H_2_	−20.9	−15.5
H_2_CO → CO + H_2_	+5.4	−27.2
CO + H_2_O → CO_2_ + H_2_	−41.2	−20.2

Thus, the utilisation of formaldehyde might become promising for applications where moderate temperatures are required as for example in the preparation of thermo-sensitive organic products or in energy storage solutions for mobile electronic devices where higher temperatures would be a disadvantage or a risk for the end-user. A limitation related to the high reactivity of formaldehyde needs to be considered in isochoric processes under dehydrogenative conditions. On the one hand, while it is beneficial to enable transfer hydrogenation reactions with appropriate hydrogen acceptor molecules under mild conditions (*vide infra*),^59–61^ in the absence of hydrogen acceptors the remaining formaldehyde may undergo reduction in the presence of hydrogen and form methanol. In this context, paraformaldehyde can be considered as a methanol surrogate (*i.e.* Ir- or Ru-cat., 25–60 °C, yields 93–95%).^62–65^ Thus, for efficient hydrogen generation it is important to release the hydrogen from the reaction vessel to increase the hydrogen yield.^28,30,66–71^

### pH-sensitivity

**Scheme 2:**
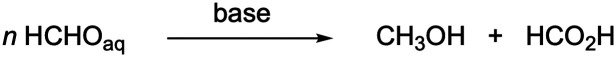
Base-promoted Cannizzaro reaction of formaldehyde which takes place as a background reaction at pH >9.5 and becomes dominant under more basic conditions (pH > 11).^65,72,73^

In addition to the thermo-sensitivity, an often underestimated issue with regards to formaldehyde conversion processes is the pH-sensitivity. Under relatively basic conditions (*i.e.* pH > 9.5), a shift of the reaction pathway may occur which results in the Cannizzaro reaction (Scheme 2),^72,73^ and at pH > 11, the formaldehyde is disproportionate to methanol and formic acid and it becomes the dominant pathway (refer to the section: Utilisation of formaldehyde and surrogates).^65^ If pH sensitivity is not considered, this phenomenon can result in misinterpretation of experimental data, where for example the generated hydrogen is assigned to aq. formaldehyde dehydrogenation, when the generated hydrogen is in fact solely derived from *in situ* formed formic acid. Thus, to prevent a classical base-promoted Cannizzaro reaction, the pH needs to be accurately buffered.^65^ A competing reaction pathway under basic conditions needs to be considered in the presence of trace amounts of carbohydrates, which may shift the reaction in the direction of the Formose reaction.^7,15–20^

**Scheme 3:**
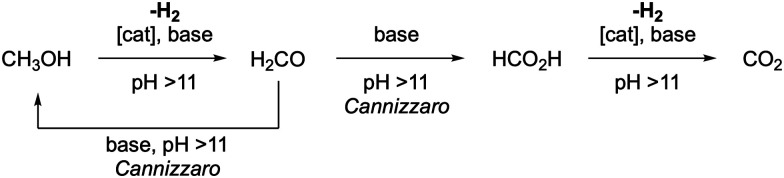
Possibility of a shift to the Cannizzaro reaction as a competing pathway during methanol dehydrogenation under strongly basic conditions.

Notably, the pH-sensitivity of formaldehyde might also be relevant for the reaction pathway of complete methanol dehydrogenation under strongly basic conditions at low dehydrogenation catalyst loadings through ligand dissociation of the coordinated formaldehyde which then subsequently undergoes the Cannizzaro reaction in the presence of base. Nevertheless, the overall H_2_ generation likely remains unchanged, since the *in situ* formed formaldehyde feeds the Cannizzaro reaction again with methanol which is subsequently converted *via* HCO_2_H into CO_2_ and H_2_ (Scheme 3). Therefore, only the discussed mechanism might be different as originally proposed for MeOH reforming under basic conditions (*i.e.* pH 14), but the outcome is likely unchanged.

### Water content

**Scheme 4:**
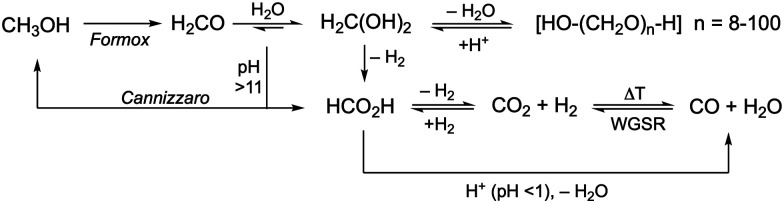
Exemplary hydration/dehydration and dehydrogenation reaction pathways of formaldehyde depending on the reaction conditions incl. water content and pH. For the FORMOX process refer to Scheme 5.

Formaldehyde, a volatile organic compound (VOC; b.p. 254 K) is commonly used as aqueous solution (formalin or formol) containing 37 wt% of formaldehyde. The formaldehyde hydrate (FAH), methanediol (H_2_C(OH)_2_) concentration in equilibrium is the dominated form (*K* ≈ 2 × 10^3^) in water and free formaldehyde is only present in low concentration (0.1%). Other species present in equilibrium in aqueous solution are oligomeric mixtures of poly(oxymethylene) glycols [HO–(CH_2_O)_*n*_–H, *n* = 1–8]. In the commercial solutions, methanol is usually added to promote stabilisation through acetal formation. The degree of polymerisation of these oligomers depends on the water content/concentration, temperature, and pH. Hence, diluted solutions likely contain lower amounts of oligomers, and in contrast, the evaporation of water from formalin results in the formation of white solid paraformaldehyde (PFA), an easy-to-handle solid formaldehyde surrogate (m.p. 120 °C, [HO–(CH_2_O)_*n*_–H, *n* = 8–100]; refer also to the section ‘production of formaldehyde and its surrogates’). PFA can be converted into the monomeric FAH through heating or under slightly acidic conditions (*e.g.* pH 5–6), where the polyacetal releases monomeric methanediol (H_2_C(OH)_2_) in aqueous solution. In organic solvents, the polyacetal opening yields non-hydrated H_2_CO for utilisation as a C1-building block in organic synthesis (*i.e.* synthesis of imidazolium salts, N-heterocyclic carbenes).^74^ Thus, depending on the targeted utilisation the water content in the reaction system should be considered to prevent undesired reaction pathways (Scheme 4).

### Methods, limitations and mitigations

Considering the above mentioned parameters in applications and planning of experiments (see also the sections below about HCHO formation and degradation), one should carefully select appropriate analytical tools for the qualitative and quantitative determination regarding formation, consumption, degradation, cross-sensitivity and selectivity of (aq.) HCHO and the respective reagents (in particular H_2_, CO_2_ and CO). For the determination for the source of HCHO or the derived products, one should consider isotope-labelling (^2^H, ^13^C, ^18^O) and analyse the samples with isotope-sensitive methods such as NMR, MS or IR for example. Isotope-labelling helps to determine the source and to exclude misinterpretation of results. Classical methods for quantification likely include titration such as Sørensen formol titration and titration with EDTA.^75,76^ And, commercial field test indicators,^77^ or digital colorimetry with smartphones (<1250 μg L^−1^) might also be suitable tools.^78^ These are examples in the analytical portfolio to complement *i.e.* the qualitative and quantitative composition of the gas phase (H_2_, CO_2_, CO, and O_2_).^28,30^ The methods for the quantitative analysis of the gas phase may include gas burettes and mass flow meters in combination with GC-TCD and mass spectrometry for further qualitative and quantitative analysis. Note here that helium as a carrier gas should be avoided for GC-TCD due to the similar conductivity to H_2_. As a carrier gas nitrogen appears to be more suitable since it allows the analysis of hydrogen and carbon dioxide accurately. In contrast, argon is relatively similar to carbon dioxide in terms of conductivity and molecular weight and therefore the separation is more challenging. For mass spectrometry, one needs to consider the low molecular weight of the gases present in the gaseous samples, as not all MS detectors are suitable for such small molecules. In case one does not have access to a GC with a TCD detector, but to a GC equipped with a FID, one should consider coupling the setup with a methaniser to enable carbon dioxide and carbon monoxide detection, since these gases are among those gases which cannot be directly analysed with a standard FID, alternatively an electron capture detector (ECD) might be considered.^79–82^ A sensitive method for formaldehyde detection in solution may include spectroscopy (*i.e.* UV-VIS) in combination with enzymatic sensors.^83^ For sample analysis and reaction monitoring, NMR and IR are also suitable but often less sensitive. Ideally the instrumental tools (*i.e.* GC; MS, IR, gas sensors flow meter, gas burette) are implemented for online reaction monitoring.

Last but not least, the experimental conditions should be well planned with the chemicals in use, and this holds true also for gaseous formaldehyde and formaldehyde solutions and the herein mentioned toxicity of formaldehyde. Therefore, experimental precautions should be well planned to avoid risks for the experimentalist. This includes experimentation in well-ventilated fume hoods, use of gloves, lab coat and safety glasses.

Experimental and analytical methodologies should consider mitigations in terms of miscibility/solubility and reactivity of the used reagents in the presence of formaldehyde. Thus, the concentration of water or other polar-protic solvents should be assessed to stabilise intermediates and control the reaction pathways. Additionally, the pH should be buffered to shift the reaction in the direction of the target product. The same holds true for the reaction temperature and pressure.

## Formaldehyde (surrogates) – production and utilisation in industries

**Scheme 5:**
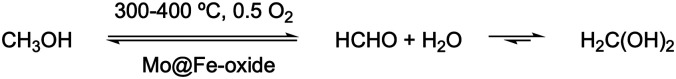
FORMOX process for the production of formaldehyde.^27,87^

The state of the art industrial production of formaldehyde through the FORMOX process (Scheme 5) with Mo/Fe-catalysts, iron molybdates,^84^ and related processes (with silver catalysts), is directly dependent on the methanol production (98 Mt per year in 2023).^85^ Likewise, formaldehyde, its derivatives and its surrogates rely on the methanol industries. Hence, about 50% of the methanol is used for the formaldehyde production.^27,59,86^ Details on process parameters and further properties can be found in the reviews under ref. 87 and further details are provided in ref. 27.

**Scheme 6:**
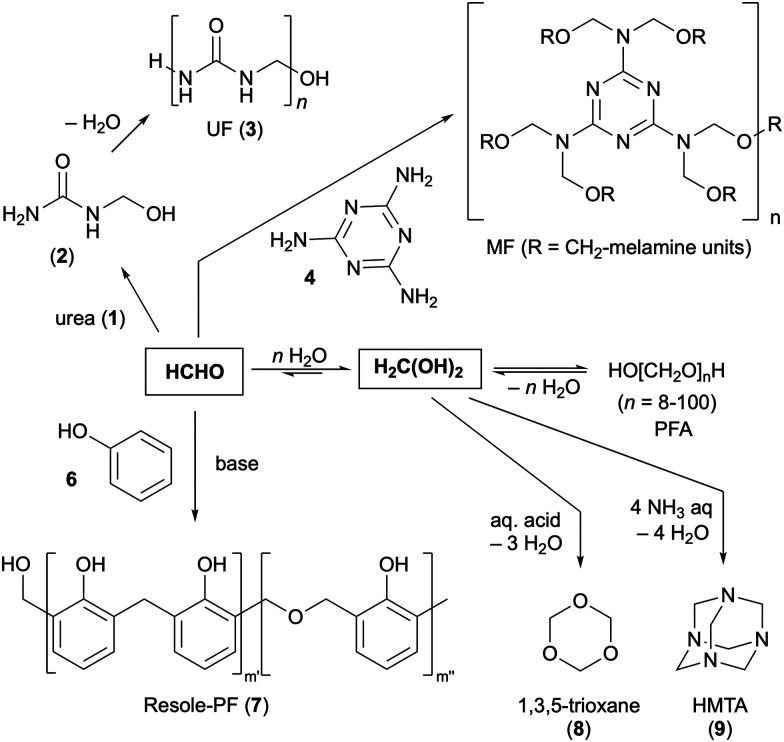
Exemplary current synthetic applications of formaldehyde for the production of formaldehyde surrogates, solid combustion fuels and resins.^29^ HMTA = hexamethylenetetramine; MF = melamine formaldehyde resin; PF = phenolic formaldehyde resins; PFA = paraformaldehyde; UF = urea formaldehyde resin.

Formaldehyde is widely used for the production of resins (*i.e.* urea-formaldehyde (UF, 3), melamine formaldehyde (MF, 5), phenol formaldehyde (PF, 7) >70% use), dyes, inks, varnishes, cosmetics, disinfectants, and other fine chemicals, as well as some hydrogen-rich surrogates such as 1,3,5-trioxane (8), paraformaldehyde (PFA) or hexamethylenetetramine (HMTA, 9; Scheme 6).^27,87^ Trioxane and PFA are formed from aq. formaldehyde through condensation and acidic dehydration respectively, while PFA may precipitate already from saturated formalin solutions by simple cooling (*vide supra*). These surrogates might also serve as an anhydrous formaldehyde source in organic synthesis.^52,88^ HMTA is readily formed by reacting aq. ammonia with aq. formaldehyde^89^ and it finds synthetic application as a base, polydentate ligand, formylation reagent, and resin hardener, to name a few. It can also be used in medicine given to its antiseptic properties and as a preservative additive (E239) in the food industries.

Albeit HMTA (*i.e.* Esbit™) and trioxane serve as solid combustion fuels in a niche market for camping or military stoves, a broader application of formaldehyde and its surrogates as an energy storage platform in the global energy sector is not significant so far. A more promising potential utilisation of formaldehyde derivatives in the energy sector targets renewable combustion fuels to complement and substitute diesel and aviation fuels (kerosene; C8–C16 aliphatic alkanes).^58,90–92^ For example, it is possible to synthesise dialkoxymethanes (oxymethylene ethers, OMEs) from the corresponding (bio-derived) alcohols and formaldehyde or CO_2_. Apart from the energy sector, formaldehyde and the before mentioned surrogates are common reagents in organic synthesis and catalysis as C1 building blocks and hydrogen donating molecules (more details are provided in the section ‘utilisation of formaldehyde and surrogates in R&D’).^27,29,52,59,74,86,88,93^ Owing to the practical aspects of solids for lab scale synthetic experimentation, PFA in particular has been frequently studied (*vide infra*).^27,52,59–62,64,66,67,69,88,94–121^

## The perspectives for the carbon, capture, utilisation and storage (CCUS) framework

### Catalytic HCHO formation and degradation

In ongoing efforts towards defossilisation and decarbonisation of the chemical industries, it is likely that the conventional production process of formaldehyde from methanol through the FORMOX process will be continued during the transition period, whilst the production of renewable methanol and renewable syngas from CO_2_ and biomass will be implemented in the first stage,^122^ and will likely continuously serve as a major feedstock for the formaldehyde industries. Nonetheless, a direct synthesis of formaldehyde from methanol, CO_2_ or syngas under milder enzymatic, thermo-, photo- or electrochemical conditions is the focus of current research.^27,51,53,59,86,123–132^ Despite the high reactivity of formaldehyde and the limiting selectivity, thermodynamics and kinetics for gas phase conversions in the case of syngas^26,51,53^ or CO_2_,^58^ it has been demonstrated that the reduction can be achieved and stabilised on the formaldehyde-level by conducting the reaction in the liquid phase in polar protic solvents such as water or alcohols,^27,53,58,86^ enabling the formation of aq. formaldehyde/formalin (methanediol) or dialkoxymethanes/oxymethylene ethers (OME), as formaldehyde surrogates. The protic polar environment is essential to shift the equilibrium of the *in situ* formed formaldehyde to the corresponding diol or acetal respectively, for the prevention of reduction to methanol.

**Scheme 7:**
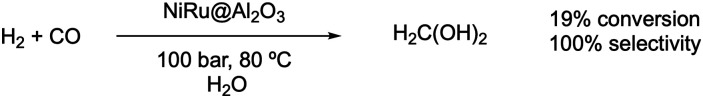
Aq. formaldehyde formation from syngas in water.^53^

Tanksale and co-workers demonstrated with a bimetallic NiRu catalyst that the endergonic (Δ*G* > +34 [kJ mol^−1^]; *K* = <10 × 10^−7^ mol^−1^) gas phase conversion of syngas to formaldehyde turns exergonic (Δ*G* <0 [kJ mol^−1^]; *K* = >1.5 mol^−1^) in water at low temperature (25–100 °C; Scheme 7) and aq. formaldehyde/methanediol is formed with 100% selectivity at a conversion level of 19% at 80 °C.^53^ Higher temperatures have been reported to disfavour the reaction in agreement with the thermodynamics. In a subsequent study, the authors reported that the efficiency can be improved by conducting the reaction in methanol as solvent and stabiliser (yield: 15.6 mmol L^−1^ g_cat_^−1^ HCHO; *T* = 90 °C; *p* = 100 bar) without affecting the excellent selectivity.^51^ The high selectivity seems favourable for flow reactions, and underlines the promising aspects to conduct the reaction under mild conditions in protic polar solvents in the absence of highly acidic or basic conditions which likely would lower the selectivity for formaldehyde. Reports on selective gas phase conversion of dry carbon dioxide to formaldehyde are scarce while the thermodynamics can be shifted significantly with water (PtC@Al_2_O_3_; dry: Δ*G* = 59.8 [kJ mol^−1^]; with water: Δ*G* = 4.91 [kJ mol^−1^]).^128,133,134^ Therefore, the ongoing research for carbon dioxide hydrogenation under enzymatic, thermo-, photo- and electrocatalytic conditions considers these limitations and tries to stabilise formaldehyde as methanediol or as oxymethylene ethers rather than free formaldehyde.

Furthermore, albeit homogeneous metal-catalysed low-temperature formation of HCHO from methanol is rare, a notable recent study on methanol oxidation to HCHO evaluated an iron complex with a hexadentate nitrogen–sulphur ligand (N_3_S_3_-tripod type ligand),^135^ which yielded a 1.3 mM concentration with 2.2 μmol catalyst under an O_2_ atmosphere at r.t. within 24 h.

### Oxymethylene ethers

Oxymethylene ethers based on methanol (DMM: dimethoxymethane, 10a) or pentanol (dipentoxymethane, 10e) for example are considered as potential complementary renewable fuels and fuel additives. These are interesting, due to their potential accessibility through biomass, CCUS and green hydrogen and to their comparable high energy density (OME: 20–23 MJ L^−1^; MeOH: *ca.* 18 MJ L^−1^) and specific energy (OME: 20–23 MJ kg^−1^; MeOH: 23 MJ kg^−1^). Also of interest, is their cleaner combustion in comparison to conventional diesel fuels and competitive production costs.^90,91,136–142^ OMEs can be synthesised through acetalisation under acidic conditions (Brønsted or Lewis acids) from: (a) alcohols and formaldehyde (surrogates such as PFA or trioxane; 2 MeOH + HCHO → H_2_C(OMe)_2_ + H_2_O), (b) through partial oxidation of methanol in alcohol (3 MeOH + 0.5 O_2_ → H_2_C(OMe)_2_ + 2 H_2_O), (c) reduction of carbon dioxide in alcohol (2 MeOH + 2 H_2_ + CO_2_ → H_2_C(OMe)_2_ + 2 H_2_O) or (d) reduction of carbon monoxide/syngas in alcohol (2 MeOH + H_2_ + CO → H_2_C(OMe)_2_ + H_2_O). The direct acetalisation of HCHO appears attractive since no metal catalysts are required for oxidation/reduction, however it strongly depends on the formaldehyde supply and the conventional FORMOX process. Albeit the direct approach with PFA and methanol proceeds in a short reaction time (equilibrium reached within less than 2 h at 60–80 °C with *i.e.* amberlyst 36), and it appears quite straightforward at low temperatures, the reaction results in product mixtures of OME, hemiacetal and trioxane, and large fractions of methanol and formaldehyde remained unreacted.^143,144^ Liquid phase extraction for the separation of the OME from the mixture is challenging due to the good solubility in both polar (water) and non-polar solvents (diesel).^143^

Hence, OME synthesis is an extension of the FORMOX process coupled with acetalisation. Current research focuses on the development of bi-catalytic solid state catalysts consisting of mixed metal and metal oxides (*i.e.* Sb, Re, V_2_O_5_, MoO_3_, Fe_2_O_3_) which combine the properties required for the oxidation of methanol and acetalisation of formaldehyde.^137^ The selectivities for OMEs are in general good (>90%) at comparable low temperatures in relation to the FORMOX process, but the conversion rates (<60%) could still be improved. In addition, Palkovits and co-workers reported on silver and copper catalysts for dehydrogenative methanol conversion in the gas phase to DMM with high selectivities (>70%) at elevated temperature (240–250 °C; *i.e.* Ag-cat.: 2 mmol_MeOH,conv_ h^−1^ g_cat_^−1^).^145–147^

Considering the feedstock requirements for OME production either from formaldehyde or coupled with methanol oxidation, the integration of such a process into the facilities of methanol and FORMOX plants is the logical consequence to extend the production capacities and the product portfolio.^138,148^

**Scheme 8:**
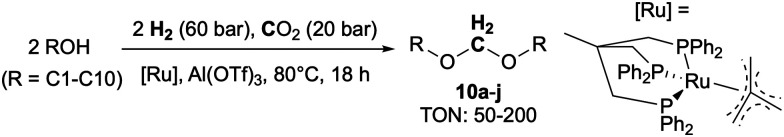
OME synthesis from carbon dioxide in alcohols (TON: turnover number).^58^

A report by Klankermayer, Leitner and co-workers^58^ demonstrated the reduction of carbon dioxide to the formaldehyde-level in alcohols (C1–C10) enabling access to dimethoxymethane (DMM 10a) and other oxymethylene ethers (OME 10b–10j). In their study, a molecular ruthenium-triphos catalyst in the presence of a Lewis acid (Al(OTf)_3_) was active to realise the formation of the OME at 80 °C within 18 h (CO_2_ = 20 bar; H_2_ = 60 bar; Scheme 8).

Further ongoing studies by Tanksale and co-workers^149–151^ evaluate ruthenium-based catalysts on β-zeolite for the hydrogenation of carbon dioxide and carbon monoxide to DMM. The highest yield for the carbon monoxide reduction to DMM was achieved with a RuNi catalyst (5.11 mmol g_cat_^−1^·L_MeOH_^−1^) at 120 °C (*p*_tot_ = 75 bar) within 48 h.^151^ While for carbon dioxide hydrogenation with a ruthenium catalyst they reported DMM yields of up to 7.42 mmol g_cat_^−1^·L_MeOH_^−1^) at 150 °C (*p*_tot_ = 75 bar),^150^ respectively 13.2 mmol g_cat_^−1^·L_MeOH_^−1^ with 100% selectivity for DMM with a recyclable ruthenium catalyst.^149^

Other early reports on the catalytic reduction of carbon dioxide to the formaldehyde-level in the presence of hydrogen donor molecules considered boranes (pinacolborane) for hydroborylation ((R_2_B-O)_2_CH_2_; Bontemps/Sabo-Etienne)^152^ and silanes (trimethylsilane) for hydrosilylation of CO_2_ ((R_3_Si-O)_2_CH_2_; Oestreich/Metsänen;^153^ Serrano/Rodriguez;^154^ Berke^155^) with the target of addressing mechanistic studies and more complex transformations in synthesis and energy conversion processes which potentially also opens perspectives for the preparation of inorganic–organic hybrid materials.^156^ These heterooxomethylene molecules may release formaldehyde upon hydrolysis for example.

## Photocatalysis for HCHO formation and degradation

The photocatalytic synthesis and formation of HCHO has been studied for several decades inspired by the role of formaldehyde in photosynthesis and the Calvin cycle.^21–24,157,158^ In addition, owing to its long known toxicity^159^ together with anthropogenic and natural HCHO emissions,^41–43,92,160–163^ there is also interest in HCHO degradation processes for the purification of exhaust gases and aqueous waste streams to improve air and water quality.^28,38,43–49,130,163–186^ However, the reports on photocatalytic formaldehyde formation from carbon dioxide or methanol are limited. Early studies, since the 1970s, reported formaldehyde as minor species along with methane, methanol or formic acid as major products.^27,187–189^ An approach with a Ru(ii) dye-sensitised TiO_2_ film for carbon dioxide reduction gave at pH 10 (*hν* >420 nm for 5 h) a mixture of HCO_2_H : HCHO : MeOH (40 : 73 : 100 with >120 μmol cm^−2^ of HCHO).^190^ The best selectivities with a photoelectrocatalytic approach to date have been obtained with a bismuth vanadium oxide anode and copper cathode, resulting in a faradaic efficiency (FE) of 85.1% (−0.9 V) for HCHO with simulated sunlight irradiation (Xe lamp, 300 W).^191^

The studies on oxidative formaldehyde formation from methanol under photocatalytic conditions give further insights about the required conditions and limitations.^132,192^ With a photocatalyst consisting of zinc indium sulfide nanocrystals (ZIS NCs) and nickel formaldehyde can be produced with 99% selectivity with high nickel loadings (6 wt% Ni precursor; 70.4 mmol g^−1^ h^−1^ HCHO), and whereas with low nickel loadings glycol (<0.5 wt 6.5–13.8 mmol g^−1^ h^−1^) is formed with 88% selectivity under UV light irradiation (365 nm).^132^ A study on the evaluation of bimetallic palladium and platinum photocatalysts PtPd@TiO_2_ under direct sunlight reported the formation of up to 0.2 μmol mg^−1^ h^−1^ formaldehyde and 44 μmol mg^−1^ h^−1^ hydrogen from methanol.^192^ The catalysts remained active over 100 h (over 20 days; 5 h sun light per day; mean value 17.5 μmol mg^−1^ h^−1^ hydrogen production).

The formaldehyde degradation to carbon dioxide may proceed under hydrogen evolution,^30^ or release of water in a gas phase reaction.^193^ PdAg@Ag core–shell nanoparticles (NPs 5 mg, dye eosin Y: 35 mg) were reported to be active for hydrogen generation yielding 0.2 mmol h^−1^ hydrogen from formalin (HCHO 0.75 mol L^−1^) under visible light and basic conditions.^194^ Using a photocatalyst consisting of NiO/BiVO_4_ ZnIn_2_S_4_ (0.3 g) and 1.5 M aq. formaldehyde under visible light (350 W Xe lamp) and basic conditions, 9 mmol g^−1^ h^−1^ of hydrogen is generated.^169^ The decomposition of gaseous formaldehyde has been studied with platinum TiO_2_ nanowires in a flow reactor under visible light irradiation with degradation rates of 86–92% of up to 50 degradation cycles in the flow system (HCHO 0.5 mg m^−3^, flow rate 50 mL min^−1^).^193^ The evaluation of the hydrogen generation from aq. formalin with CuNi@C/TiO_2_ nanoparticle photocatalysts under basic conditions yielded 254.6 mmol g^−1^ h^−1^ hydrogen.^129^

### Electrocatalysis and fuel cells (FC) for HCHO formation and degradation

Highly selective electrochemical reduction of carbon dioxide to formaldehyde remains a rarity due to the stability and reactivity of formaldehyde.^27,126,128,195,196^ Nakata and Einaga studied carbon dioxide reduction to formaldehyde with boron-doped diamond electrodes and platinum counter electrodes (−1.0 V to −1.9 V *vs.* Ag/Ag^+^) with a maximum faradaic efficiency for formaldehyde of 74% at −1.7 V over 20 h in a methanol electrolyte.^195^ In these studies, they also used seawater as an electrolyte yielding 36% aq. formaldehyde (7.5 mM h^−1^) and 62% when 0.1 M aq. NaCl was used as an electrolyte. In a study by Nam and co-workers, Sn and Pt electrodes together with Lewis acids were evaluated for carbon dioxide reduction and methanol oxidation with a focus on the formation of formaldehyde and its derivatives DMM and PFA.^126^ They demonstrated faradaic efficiencies of 50% (cathode) and 90% (anode) with a charge of 200C, for the combined production PFA and DMM.

In contrast to the studies mentioned above in this section, Robert and co-workers^197^ explored the electrochemical reduction of carbon monoxide. In their studies, they used electrodes modified with multiwalled carbon nanotubes (MW-CNTs) and an immobilised cobalt phthalocyanine complex. Optimised conditions in this study were found to be pH 12 and 10 °C, while the Cannizzaro reaction does not play a role under these conditions (potential, pH and time scale) and the formate concentration remained below 1%. The best selectivity for HCHO (FE 17.5%; TON = 349; TOF = 0.2 s^−1^) was obtained with a maximum partial current of *E* = −0.650 V_RHE_ and current density of 0.64 mA cm^−2^.

Electrocatalytic oxidation of dry methanol to formaldehyde with platinum electrodes have been reported by Sasaki and Nagaura with Faraday efficiencies to formaldehyde of 70% (0.1 M NaOMe in MeOH; 2 h, current density of 3.3 mA cm^−2^),^198^ respectively, by Mechler and co-workers with a Faraday efficiency of 90% for a current density of 100 mA cm^−2^.^123^ The product concentration of the latter conditions is equal to 7.7 g L^−1^ after 30 min (production rate of 17 mol h^−1^ m^−2^). In a different approach, Garcia and co-workers^125^ studied the electrochemical oxidation of methanol to HCHO with a polymer-bound 2,2,6,6-tetramethylpiperidinyloxyl radical (TEMPO) which is known for selective oxidation of alcohols to aldehydes. The polymer-modified glassy carbon electrode contains a TEMPO with a pyrrole backbone. The reactions were conducted with methanol as the solvent and reactant in the presence of different bases where among the tested bases, lutidine (5 mM concentration) gave the best results at a potential of 1.0 V with a maximum FE of 97.5% and a TON of 1250.

In addition to the demonstrations for electrocatalytic formaldehyde formation from carbon dioxide and methanol, it has been also evaluated for the decomposition under hydrogen release and in formaldehyde-fed fuel cells.^44,59,181,186,199–217^ Formally the oxidative electrochemical degradation of formaldehyde proceeds at the anode (HCHO + H_2_O → CO_2_ + 4 H^+^ + 4 e^−^) and the sacrificial component is reduced at the cathode (*i.e.* aerial O_2_: O_2_ + 4 H^+^ + 4 e^−^ → 2 H_2_O). Typical studied electrocatalysts for such fuel cells include metals, alloys and metal oxides (*i.e.* Ru, CuPd, Ce, Cu, CuO, NiO, and TiO_2_) or enzymes/bacteria for formaldehyde degradation in microbial fuel cells.^44,186,202–204,206–208,210,212,214,215^ Even though the HCHO degradation in microbial fuel cells is interesting, so far the efficiency is limited with reports and samples with 200 ppm of HCHO requiring 152 h to reduce the concentration by 89%.^204^ An example for the evaluation of a fuel cell for formaldehyde degradation under CO_2_ evolution with a TiO_2_-photoanode and a Cu_2_O photocathode in a fuel cell,^206^ reached a current of 1.2 mA cm^−2^ with a voltage of 0.58 V under sunlight illumination (100 mW cm^−2^) at room temperature. The setup used a proton exchange membrane (PEM), 0.5 M sodium sulphate as the electrolyte, and 1 M aq. formaldehyde and pure oxygen for the oxygen reduction reaction (ORR). In a more recent study by Fu and co-workers, it was demonstrated that a formaldehyde fuel cell is capable of operating without carbon dioxide evolution.^202^ The terminal oxidation product is formate in this case and the electrochemical oxidation of formaldehyde to formate (53 mol) proceeds under hydrogen evolution (0.62 N m^3^ with a faradaic efficiency of 100% between 0–0.3 V_RHE_) per 1 kWh of generated electricity (voltage: 1 V, peak power density: 350 mW cm^−2^). The study used an anion exchange membrane (AEM), metallic copper foam/nanosheets as anodes, platinum on a carbon cathode and 1 M KOH as the electrolyte. The authors analysed the role of a possible Cannizzaro reaction, and concluded that it is negligible under these conditions. In a follow-up study on aq. formaldehyde, the same group reported higher hydrogen yields along with a lower amount of formate and generated electricity.^186^ A study by Chen and co-workers^203^ used a PdCu alloy as the catalyst material. The oxidation of formaldehyde at 0.2 V results in a current density of 50 mA cm^−2^ and the current remains stable over 100 h and the best results reached an open-circuit voltage of 0.9 V and a peak power density of 100 mW mg_Cu_^−1^. In a different context, an HCHO FC electrode with amorphous nickel tungstate nanoparticles was evaluated as a sensor for residue HCHO,^207^ and a detection limit of 3.6 μM was determined.

### Bioremediation & biomanufacturing

In addition to chemical means that deal with issues related to formaldehyde as a pollutant, tailor-made biological systems can also offer an attractive approach to the selective decomposition for formaldehyde. Despite the general cytotoxicity of the C_1_ aldehyde, a number of microorganisms have been identified that facilitate the metabolic oxidation to CO_2_ and certain strains can be employed for rather effective removal of the pollutant.^171,218^ While some species tolerate impressive concentrations of the toxic aldehyde,^219^ typically, bioremediation is performed with immobilised whole cell systems.^220–222^ The functional principles of the overall metabolic pathways in formaldehyde-degrading bacteria and archaea goes beyond the scope of this tutorial, yet knowledge of the key enzymes such as formaldehyde dismutase^223^ or formaldehyde dehydrogenase (FAldDH),^224,225^ which are directly involved in the conversion of the aldehyde substrate,^226^ can inform the design of *in vitro* enzymatic,^227^ biomimetic,^65^ or chemoenzymatic C_1_ conversion pathways.^228,229^

Besides, the same knowledge of crucial C1-converting enzymes can essentially also form the basis for the biomanufacturing of formaldehyde as a future production platform of this indispensable chemical building block. Particularly, CO_2_ represents naturally a highly attractive raw material where formate dehydrogenases as well established enzymes enable the reductive activation of the greenhouse gas.^230–232^ While *in vitro* multi-enzyme cascades for CO_2_ fixation have so far mainly focused on methanol production,^233–235^ recently the first successful formaldehyde biomanufacturing reports have appeared. Liu and co-workers established a photo-enzymatic CO_2_ fixation leading to formalin concentrations up to 4.1 mM. The recycling of the NADH cofactor that both FDH and FaldDH require is accomplished through a complex UV light-driven electron transfer chain including a homogenous rhodium complex and TiO_2_ as a photocatalyst where EDTA acts as terminal reductant.^236^ A purely biocatalytic cascade on the other hand was described by Huang and co-workers, which could be employed with NADH as a stoichiometric reducing agent or with a phosphite/phosphite dehydrogenase recycling system.^225^ The formaldehyde yields of this *in vitro* reduction, however, remained significantly lower.

In addition to these reductive pathways based on CO_2_ fixation, methanol can also serve as a raw material for formaldehyde production. As a consequence of the diversity of natural methylotrophs, a range of methanol-oxidizing enzymes is available, including NAD-dependent methanol dehydrogenases (MDH), PQQ-dependent MDHs (PQQ: pyrroloquinoline quinine), and O_2_-dependent alcohol oxidases (AOx).^237^ While the former enzymes play central roles in the pathway optimization for microbial methanol valorisation, alcohol oxidases are particularly attractive enzymes as they operate without the need for any cofactor recycling systems and air as the sole oxidant. The aerobic dehydrogenation delivers aqueous formaldehyde alongside hydrogen peroxide, the latter being typically decomposed *in situ* by the addition of catalase in order to reduce the risk for oxidative deactivation of the oxidase enzyme.^238,239^

## Utilisation of formaldehyde and surrogates

### Organic synthesis with formaldehyde

**Scheme 9:**
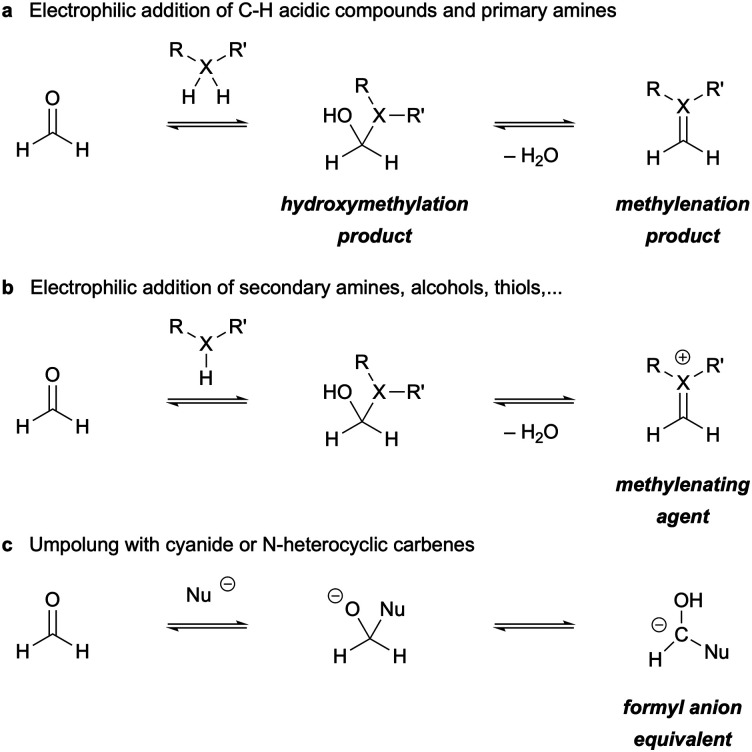
General electrophilic reactivity of formaldehyde with C-nucleophiles, amines, alcohols and thiols (a and b); as well as its umpolung in the presence of nucleophilic catalysts (c). R′ = carbon substituent or lone pair.

In the context of organic synthesis, formaldehyde exhibits the same kind of electrophilic carbonyl reactivity as its higher homologues, while lacking the nucleophilic alpha-reactivity of other, more complex carbonyl compounds. The characteristic aldehyde reactivity can lead to a number of different species, some stable, some reactive intermediates. The reaction with *C*-nucleophiles and primary amines proceeds through addition to the formaldehyde's carbon under the formation of a hydroxymethylated product (Scheme 9(a)), that, depending on the electronic nature of the nucleophile, can be isolated or will further eliminate water to yield a methylenated product (*i.e.* a terminal olefin or imine).

On the other hand, the reaction with other heteroatom nucleophiles such as secondary amines or alcohols, typically in the presence Brønsted or Lewis acids, will take a similar addition–elimination pathway that leads to highly electrophilic iminium or oxonium species, respectively (Scheme 9(b)). These intermediates are readily attacked by other nucleophiles so that formaldehyde acts eventually as a methylene bridge. Lastly, in the presence of nucleophilic species such as cyanide or N-heterocyclic carbenes, formaldehyde can undergo an umpolung reaction where a carbanionic intermediate is formed which functions as a nucleophilic formyl transfer agent (Scheme 9(c)).^240^ While the umpolung pathways have found only very limited application in traditional organic synthesis beyond formose-type formaldehyde oligomerizations (mainly due to Cannizzaro disproportionation side reactions,^16^ we will revisit this reaction mode later during the discussion of organocatalytic methodologies.

**Scheme 10:**
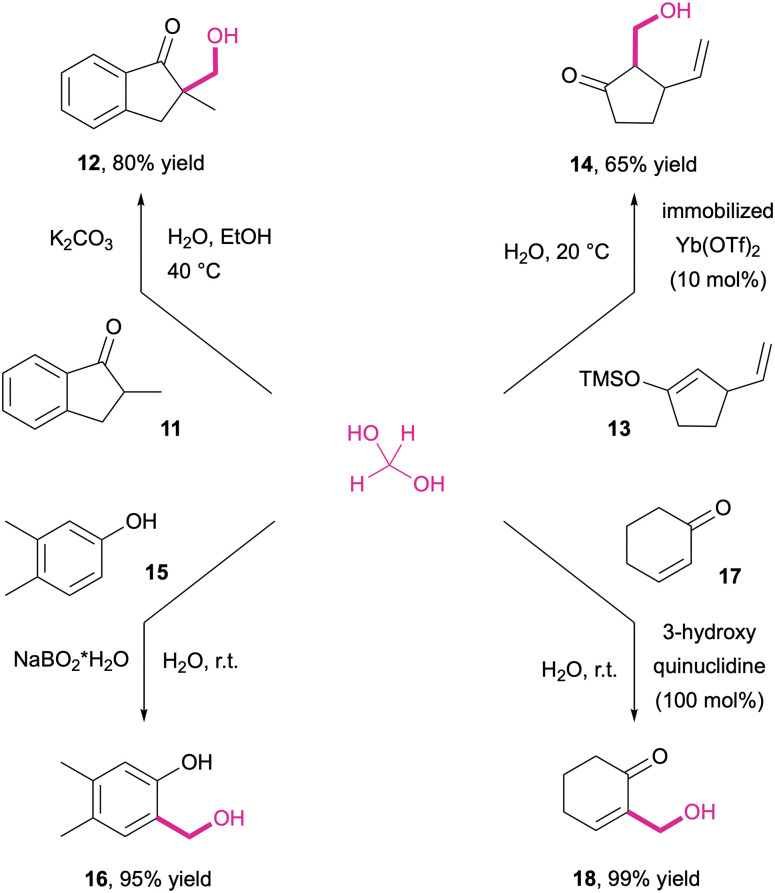
Hydroxymethylation of carbon-centred nucleophiles Brønsted base-catalysed (top left and bottom left); Lewis acid catalysed (top right); Lewis base-catalysed (bottom right).

The synthetic application of the hydroxymethylation reactivity can be widely observed with a broad range of C-nucleophiles. Like most other aldehydes, formaldehyde also readily engages in aldol-type C–C bond forming reactions. Due to the relatively high carbonyl reactivity of the C1 aldehyde, however, the selective reaction with only one equivalent of formaldehyde is challenging,^241^ as sparsely substituted ketones, esters and other enolate precursors often suffer from exhaustive per-hydroxymethylation.^242^ Hence, direct hydroxymethylation with aqueous formalin is mostly limited to α,α-disubstituted carbonyl compounds such as α-methylindanone 11 where this selectivity issue is non-existent, which delivers the β-hydroxy products 12 in generally high yields (Scheme 10, top left).

In order to achieve a selective mono-hydroxymethylation of less substituted ketones, the Mukaiyama protocol based on the Lewis acid activation of aqueous formalin in the presence of silyl enol ethers (13) poses a valid alternative (Scheme 10, top right).^243^ Also non-sp^3^ carbon nucleophiles such as phenols (15) or enones (17) can be employed. The former is for example accomplished by means of metaborate as a reaction mediator (Scheme 10, bottom left), facilitating selective ortho-hydroxymethylation (16) without the risk of polymerization.^104^ The Morita–Baylis–Hillman reaction between enones/enoates and formalin proceeds in the presence of a stoichiometric amount of Lewis-basic N-heterocycles such as 1,4-diazabicyclo[2.2.2]octane (DABCO) or 3-hydroxyquinuclidine (Scheme 10, bottom right).^244^

**Scheme 11:**
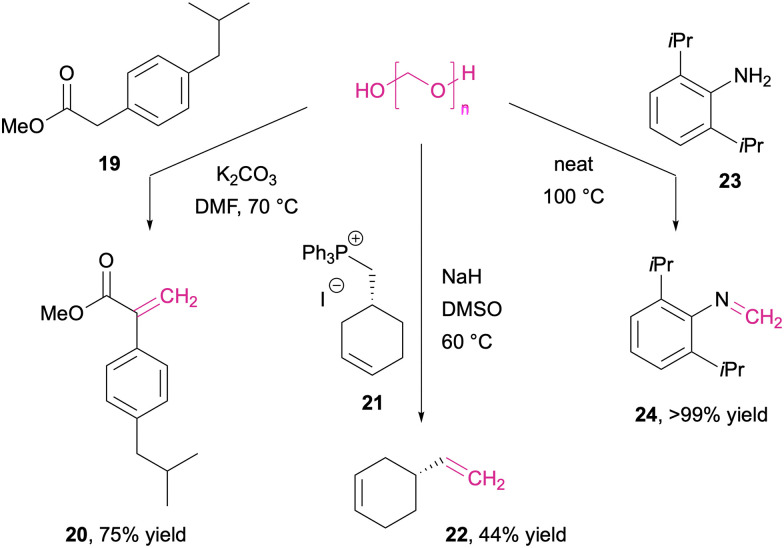
Olefinations and imine synthesis with paraformaldehyde. Brønsted base-catalysed (left and centre), thermal olefination under neat conditions (right).

With a slightly different set of *C*-nucleophiles, the reaction with formaldehyde can also be diverted to α-methylenations. Pre-nucleophiles carrying a CH_2_ moiety between two activating groups such as carbonyls, nitriles, arenes and/or nitro groups, typically do not stop on the hydroxymethylation stage but rather react in Knoevenagel-type condensation (20) with paraformaldehyde under basic conditions (Scheme 11, left).^245^

Without the intrinsic reactivity of electron-withdrawing functionalities, simple unsaturated terminal alkenes (22) are also accessible with paraformaldehyde when phosphonium ylides (21) are employed as nucleophiles in Wittig olefinations (Scheme 11, centre).^246^ In addition to the polymeric anhydrous formaldehyde, surrogates such as hydroxymethyl triphenylphosphonium salts or *N*-hydroxymethyl phthalimide have also found application in transformations where strong bases and anhydrous conditions are required.^247^

Different from the hydroxymethylation that is generally not employed with nitrogen nucleophiles other than the reagent preparation of these surrogates such as the above mentioned *N*-hydroxymethyl phthalimide, formaldehyde condensation has been observed and the corresponding C1 imines could be isolated in cases where the nitrogen nucleophile (23) provides sufficient steric protection of the highly reactive Schiff base (Scheme 11, right).

**Scheme 12:**
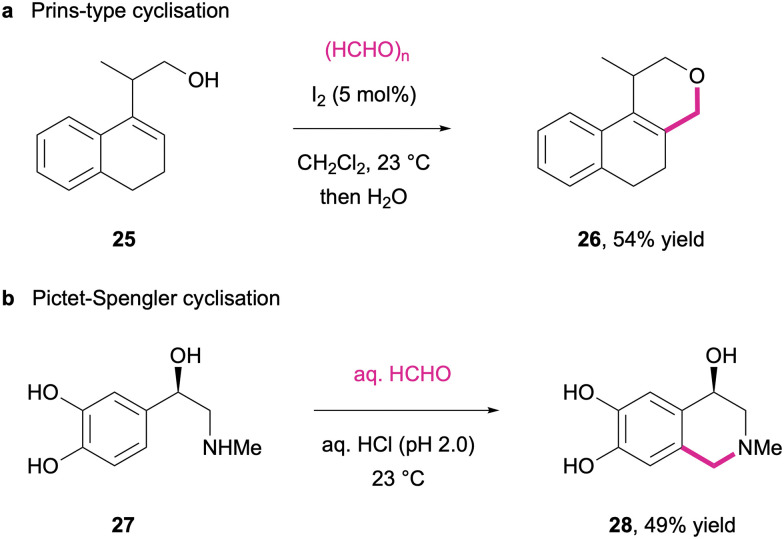
Representative cyclisation reactions *via* formaldehyde-derived oxonium (a) and iminium (b) intermediates.

While formaldehyde as one of the smallest organic molecules usually does not contribute much to the complexity of the synthetic products, the generation of formaldehyde-derived iminium/oxonium intermediates in particular offers highly interesting pathways for cyclisation reactions and especially those in multi-component reactions. Both alcohol-derived oxonium intermediates as well as amine-derived iminium salts have been successfully employed to intermolecularly link the heteroatom functionalities through a methylene bridge with carbon-centred nucleophiles. Thus, oxygen-heterocycles are obtained through reaction of formaldehyde with simple alkenes^248^ or homoallylic alcohols (25) through Prins-type cyclizations (Scheme 12(a)).^249^ In a similar fashion, biogenic amines such as epinephrine (27) can be used in Pictet–Spengler cyclizations where the formaldehyde-derived iminium species engages in electrophilic aromatic substitutions to yield tetrahydroisoquinoline derivatives (28) (Scheme 12(b)).^250^

**Scheme 13:**
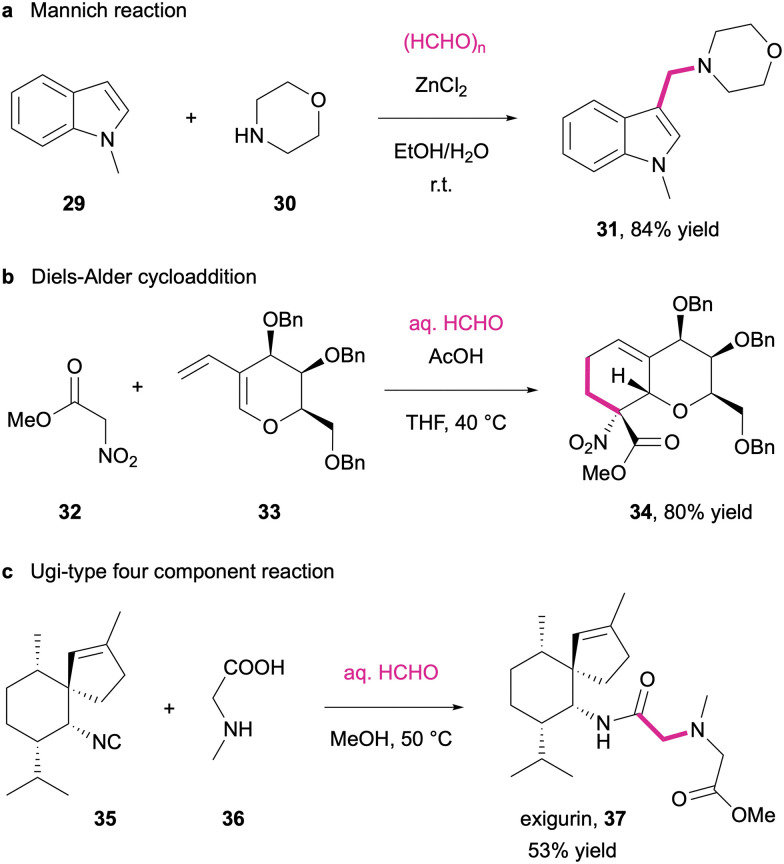
Representative multi-component reactions using formaldehyde as the C1 linker element. (a) Mannich reaction; (b) Diels–Alder cycloaddition; (c) Ugi-type four component reaction.

The high carbonyl reactivity and ease of formation of oxonium intermediates, iminium salts or olefinic Knoevenagel products in complex reaction mixtures has led to a high popularity of formaldehyde and its surrogates as a critical element in multicomponent reactions.^251^ The application scope is very wide and has been nicely compiled in a critical review by Jérôme and co-workers.^252^ Particularly, the reaction between formaldehyde-derived imines/iminium intermediates and *C*-nucleophiles has been a popular target of organic method development, including Petasis and Mannich reactions.^253^ For example, nucleophilic N-heterocycles such as indole, pyrrole and the likes find frequent application in Mannich-type reactions where secondary amines and formaldehyde in the presence of a Lewis acid deliver aminomethylated products (31) in high yields (Scheme 13(a)).^254^ But also Knoevenagel condensations with formaldehyde as an electrophile offer interesting opportunities for multicomponent reactions. Jayakanthan *et al.* used *in situ* condensation between formalin and methyl nitroacetate (32) to generate the corresponding nitroacrylate in the presence of a sugar-derived diene (33). Spontaneous [4+2]-cycloaddition trapped the reactive enoate and delivered bicyclic carbohydrate derivatives (34) in high yield and excellent diastereoselectivity (Scheme 13(b)).^255^ Moreover, a formaldehyde-based multicomponent reaction was also established as a final synthetic step in the preparation of the marine natural product exigurin (37).^256^ In a four component Ugi reaction, a terpenoid isonitrile (35) is reacted with the iminium intermediate resulting from the condensation of sarcosine (36) with formalin, and ring opening of the thus formed cyclic mixed anhydride by the solvent methanol delivers the sponge metabolite in 53% yield (Scheme 13(c)).

### Organocatalysis with formaldehyde

The previously discussed basic application patterns of formaldehyde in organic synthesis fall right within the core application of many organocatalytic methodologies that often deal with the activation of carbonyl compounds. Hence, many of these methods were also found to be useful when it comes to reactions between a range of carbonyl compounds and formaldehyde. Here, organocatalytic activation was proven to provide certain benefits over traditional methods as exceptionally mild conditions help to overcome challenges related to selectivity posed by the high reactivity of the C1 aldehyde.

**Scheme 14:**
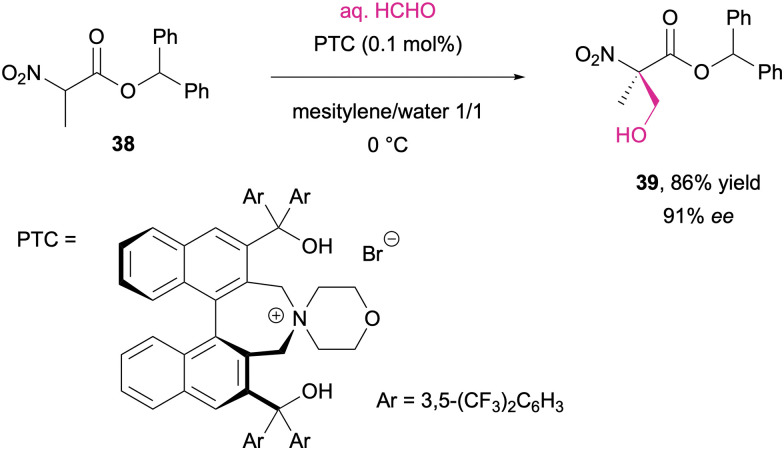
Asymmetric hydroxymethylation of α-nitroesters through phase transfer catalysis (PTC).^258^

The hydroxymethylation pathway illustrates these issues well, as the small footprint of formaldehyde renders it more challenging than other electrophiles in asymmetric aldol additions while also being prone to elimination and double hydroxylation side reactivities.^257^ Maruoka and co-workers reported on a system employing phase transfer catalysis for the enantioselective hydroxymethylation of α-nitroesters where aqueous formalin was utilized in a biphasic reaction medium. With the aid of an axially chiral *C*_2_ symmetric ammonium salt under base-free conditions, good optical purities were achieved, and the products were further transformed into α,α-disubstituted serine derivatives (Scheme 14).^258^

**Scheme 15:**
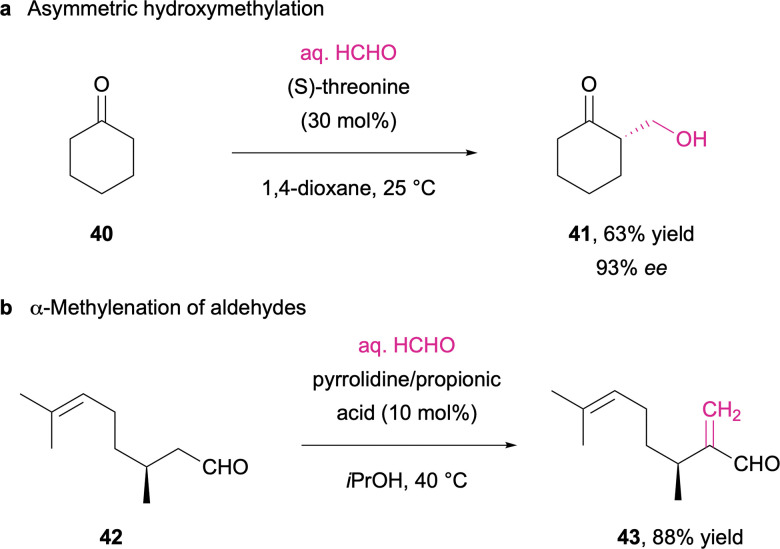
Aldol addition (a) and aldol condensation (b) reactions between enolisable carbonyl compounds and formaldehyde.^259,260^

Similarly, the enamine catalysis pioneered by List and MacMillan was also examined as a tool for the selective aldol chemistry of formaldehyde. In a study by Takabe and co-workers, the amino acid threonine was identified as the most effective catalyst, and the selective mono-hydroxymethylation of cyclic ketones (40 to 41) was accomplished in moderate to high enantioselectivities (42 to 43; Scheme 15(a)).^259^ Slight modification of the reaction conditions on the other hand leads to an aldol condensation pathway where secondary amine catalysts in presence of a Brønsted acid cocatalyst facilitate the α-methylenation of aldehydes (Scheme 15(b)),^260^ or the γ-methylenation of enals.^261^

**Scheme 16:**
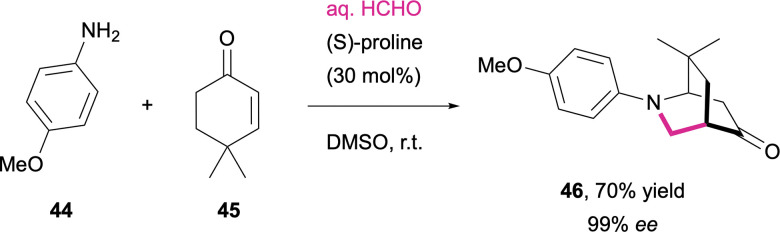
Proline-catalyzed three component enantioselective aza-Diels–Alder cycloaddition.^262^

The amino acid-mediated activation of ketones and enones can further be extended to multicomponent reactions. Sundén *et al.* illustrated this approach in an asymmetric aza-Diels–Alder cycloaddition, where proline-derived dienamines recombine with *in situ* formed methylene imines, leading to the formation of optically pure bicyclic N-heterocycles (46; Scheme 16).^262^

**Scheme 17:**
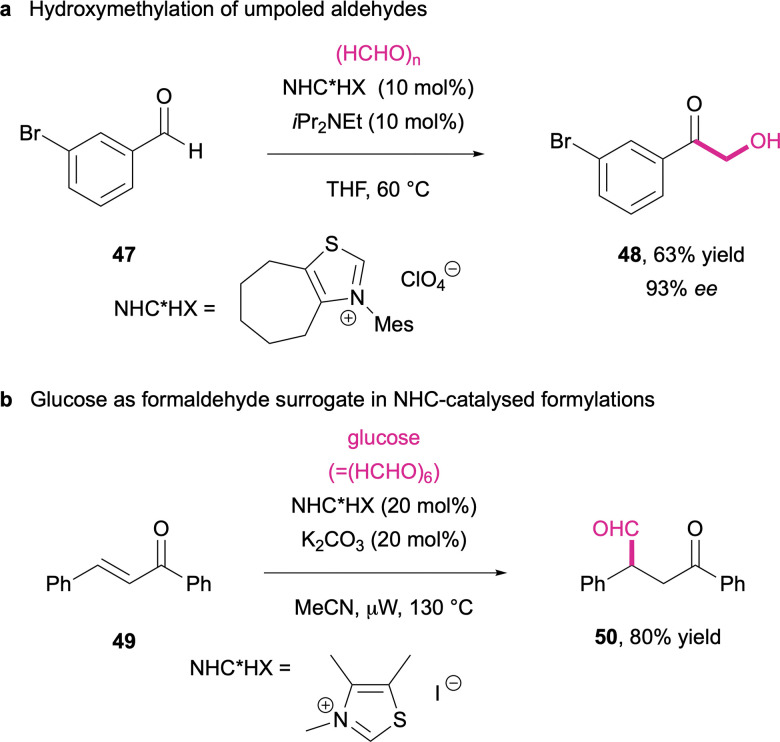
NHC-catalysed C–C bond formation through acyl anion equivalents. (a) shows the hydroxymethylation of umpoled aldehydes and (b) the use of glucose as a formaldehyde surrogate for an NHC-catalysed formylation.^265,266^

Organocatalysis has so far also proven to be the best solution for any attempts to conduct umpolung of formaldehyde or its surrogates in order to transfer formyl groups in a nucleophilic fashion. However, whenever direct umpolung of formaldehyde was attempted through the use of nucleophilic N-heterocyclic carbenes (NHCs), either self-condensation to higher carbohydrates was observed,^263^ or cross acyloin reactions took place where a secondary alcohol was selectively converted to the nucleophilic Breslow intermediate.^264^ The latter leads to an alternative hydroxymethylation pathway where aldehydes are activated by thiazolium-derived nucleophilic NHCs and add to the electrophilic formaldehyde, furnishing α-hydroxyketones (48) (Scheme 17(a)).^265^

So far, the only way to effectively generate nucleophilic Breslow intermediates of formaldehyde utilises higher carbohydrates as formaldehyde surrogates. Here, the stepwise NHC-catalysed degradation of sugars reversibly leads to the nucleophilic formyl anion equivalents. As demonstrated by Chi and co-workers, glucose can be employed as “formaldehyde hexamer” and in the presence of simple trimethylthiazolium salts and K_2_CO_3_, formyl transfer through Stetter reaction with enones (49) is achieved in high yields (Scheme 17(b)).^266^

### Formaldehyde and biocatalysis

The presence of formaldehyde in biocatalytic reactions is rich and varied, as this most simple aldehyde can be found steadily both in living systems and in laboratory-based environments – with dramatically different effects and applications.

### Routes to and from formaldehyde

**Scheme 18:**
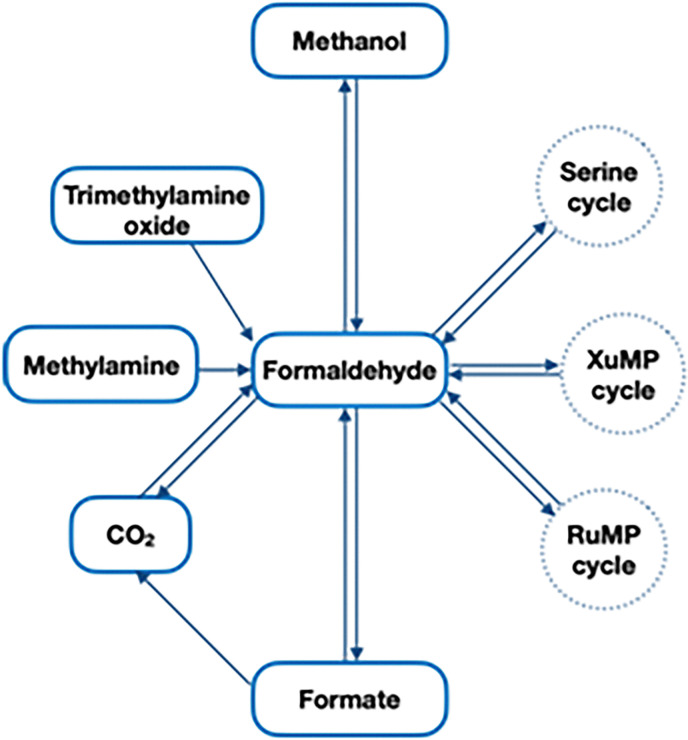
Summary of the most common routes to and from formaldehyde, both *in vivo* and *in vitro*.

Formaldehyde is abundantly found in biological pathways, being introduced by external sources or endogenously produced *in vivo*.^267^ Not only HCHO formation is the result of catabolic oxidation of methanol,^268^ but it is also involved in crucial pathways, such as the ribulose monophosphate (RuMP) and the xylulose monophosphate (XuMP)^269^ or the serine cycles.^270^ These natural routes have served as an inspiration to expand the role of formaldehyde for hydrogen storage and production through biomimetics.^228,229^ Alternatively, some bacteria are also known to produce formaldehyde from methylamine through methylamine dehydrogenase (MADH)^271^ or from trimethylamine oxide through a trimethylamine-*N*-oxidase (TMAOase),^272^ amongst others. From a man-made perspective, the conversion of CO_2_ to formaldehyde has been exploited both chemically and enzymatically;^27,273^ and the CO_2_-derived formaldehyde could also be further condensed to generate C3 compounds.^274^ Most of these processes, especially those which are part of larger and more intricate pathways, are – or can be made – reversible; rendering this C1-aldehyde both a product of anabolic construction and a substrate for catabolic degradation (Scheme 18).^65,269,270,275^

### Endogenous formaldehyde (“formaldehyde *in vivo*”)

Given its ubiquitous presence in nature, it is of no surprise that formaldehyde rises with a key physiological position: that of a metabolic regulator. In this sense, FA has been proven to be of crucial importance for epigenetic dysregulation and genome disability, by inhibiting the biosynthesis of S-adenosyl methionine (SAM) in cells – a development that is of relevance for serious conditions such as fatty liver disease and cancer.^276,277^

This key role as a regulator is partly because HCHO is highly toxic and carcinogenic,^159,278^ having been established that formaldehyde and its metabolism are directly linked to DNA damage repair and genotoxicity^279,280^ and to protein–DNA crosslinking.^281^ In this line, many studies have been conducted to establish the chemistry of formaldehyde with amino acids and proteins.^282–284^ Formaldehyde's high toxicity *in vivo* has also led to the development of natural detoxification systems^285^ and fuels the establishment of these highly valorisable cycles,^286^ as different metabolic pathways have evolved to circumvent such eventuality, and in turn, transform it into more valuable substrates.^287,288^

### Exogenous formaldehyde (“formaldehyde *in vitro*”)

**Scheme 19:**
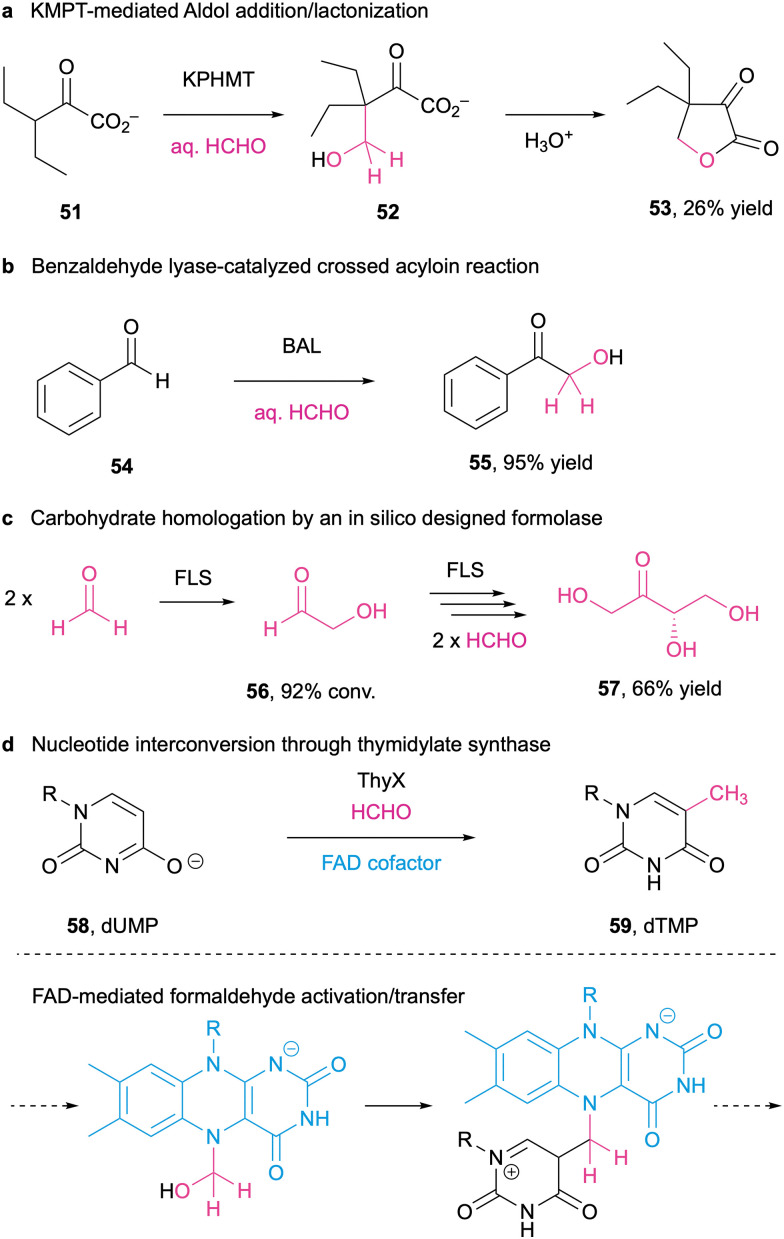
Formaldehyde in biocatalysis. (a) 3-methyl-2-oxobutanoate hydroxymethyltransferase (KPHMT) is able to incorporate formaldehyde to produce 2-oxolactones; (b) benzaldehyde lyase (BAL); (b) ThDP-dependant enzyme, catalyses C–C bond formation to yield 2-hydroxy-1-phenyl-ethan-1-one; (c) formolase (FLS) is a computationally-designed enzyme that allows the development of a novel one-carbon assimilation pathway; (d) reductive methylation of deoxyuridylate (dUMP) into deoxythymidylate (dTMP) from formaldehyde, using thymidylate synthase (ThyX).^290–294^

In truth, formaldehyde has been gaining a more prominent role in the biosynthesis of fine chemicals.^289^ Its unique reactivity and versatility set it apart from other C1 scaffolds, as is the fact that FA is an intermediate of methylotrophic microorganisms. In this way, the exploration of biocatalytic routes to exploit (chiral) transformations of formaldehyde has been mostly defined in three ways: C–C aldolase bond formation (Scheme 19(a)),^290^ umpolung activation by thiamine diphosphate dependent enzymes (ThDPs; Scheme 19(b)),^291^ and a formolase reaction (Scheme 19(c)).^292–294^ Indirectly speaking, formaldehyde is also used by thymidylate synthase (ThyX) as a direct methylene donor of deoxyuridylate (dUMP) in folate-dependent reactions, which are a critical step toward DNA biosynthesis (Scheme 19(d)).^295^

### Photoredox catalysis

**Scheme 20:**
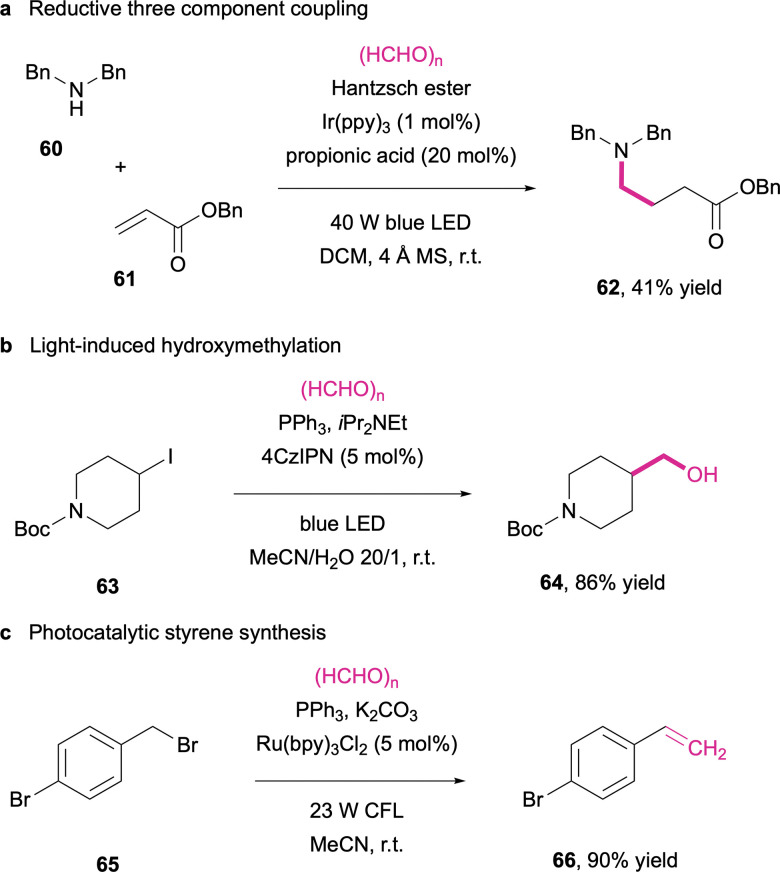
Aminomethylation (a), hydroxymethylation (b) and olefination (c) based on visible light-driven activation modes.

More recently, photoredox catalysis has experienced a veritable boom as a versatile new tool in the organic chemist's toolbox.^296^ The development of organic or organometallic dyes has opened up reaction pathways that in many ways complement the other catalytic approaches through mild light-induced generation of reactive radical species.^297^ Formaldehyde itself has not yet been a major focus in the methodology exploration based on photoredox catalysis, however, the C1-aldehyde occasionally finds recognition as part of substrate scope investigations as illustrated by the reductive aminomethylation of butyl acrylate (61) in the presence of an iridium dye (Scheme 20(a)).^298^ So far, two studies have explicitly taken a closer look at formaldehyde as a C_1_ building block, both in combination with alkyl halides (63) and triphenylphosphine. Leonori and co-workers reported on the use of the isophthalonitrile dye as a mediator in a broadly applicable dehalogenative hydroxymethylation (Scheme 20(b)),^299^ while Jiang *et al.* observed under slightly different conditions, using a ruthenium-based photoredox catalyst and an inorganic base, the conversion of benzylic bromides (65) into the corresponding styrene derivatives (66) (Scheme 20(c)).^300^

### Metal-catalysed introduction of CH, CH_2_ and CH_3_ entities

As outlined above, formalin and PFA are well established in classical organic synthesis for the introduction of C1-entities (*i.e.* in aldol condensation, Baeyer diarylmethane synthesis, and Blanc Mannich, Petasis, Prins–Kriewitz, Tiffeneau reactions). In time, such protocols and substrate scopes were extended through metal catalysis to enable new protocols or give new insights for methylation (excerpts in Table 2), hydroxymethylation, alkynylation, and synthesis of allenes among others.^101,119,301–312^

**Table 2:** Examples of methylation catalysed by molecular metal complexes

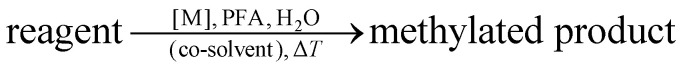						
Entry	Cat.	*T* [°C]	Reagent	Product	[%]	Ref.
1	Rh^a^	180	Ketone	α-Methylated ketone	≤54	307
2	Rh^a^	180	Amine	*N*-Methylated amine	≤92	308
3	Co^a^	120	Amine	*N*-Methylated amine	≤98	309
4	Ru	60	Amine	*N*-Methylated amine	≤98	119

a Under CO pressure.

Notably, early examples for methylation reactions with rhodium and cobalt catalysts, performed the reactions under CO pressure to enable the formylation followed by reduction to the methyl entity.^307–309^ The more recent study on *N*-methylation with ruthenium catalysts allowed for the conversion through subsequent imine formation followed by transfer-hydrogenation of the *in situ* formed N=CH_2_ entity to N–CH_3_. The protocol is also applicable in a biphasic solvent system which allows catalyst recycling and product separation.^119^ Interestingly, allylic amines are accessible through Pd-catalysed coupling of alkenes with amines in the presence of PFA.^313^ Allylic alcohols are accessible in moderate to good yields (39–86%) for example through Ru-, Ni- or Ir-catalysed hydroxymethylation of dienes, allenes and alkynes with PFA as the C_1_-donor usually in the presence of protic solvents or additional terminal reductants to improve the yields (*i.e.* water, alcohol, formic acid).^95,109,116,314–318^ And, propargylic alcohols can be synthesised in good yields from terminal alkynes and PFA with silver or copper catalysts.^97,305^ The hydroxymethylation of substituted 1,4-dienes gives access to the corresponding coupling products on the C1 (≤63%) or C4 (≤71%) position applying a Ni-catalyst, while with ruthenium the coupling occurs at the C2 (≤74%) or C3 (≤80%) position.^314^ The hydroxymethylation of substituted alkynes occurs preferably at the less hindered side (≤81%) with nickel while with ruthenium the isomer with the bulkier substituent (≤85%) is accessible.^95^ Moreover, hydroxymethylation of aromatic ring systems with PFA has been demonstrated with azaindoles in the presence of a ruthenium catalyst giving the products in good yields (up to 83%).^101^ The *ortho*-selective ruthenium-catalysed hydroxymethylation of benzoic acid amides with PFA yields phthalides after subsequent lactonisation (up to 72%).^121^ Interestingly, the treatment of pyridinium salts with PFA, magnesium methoxide and potassium iodide in the presence of a ruthenium catalyst leads to dearomatisation and regioselective hydroxymethylation.^319^ In a different context, hydroxymethylation with PFA of α-cyanocarboxylates occurs with (chiral) rhodium and palladium catalysts, while with gold catalysts dihydrooxazoles are formed in moderate to good yields and enantioselectivities.^320–322^

In the broader field of organic synthesis, aq. formaldehyde and PFA find diverse applications in the synthesis of heterocycles and functionalisation of aromatic compounds. Prominent examples include the synthesis of imidazolium salts which are of interest for the generation of stable Arduengo-type carbenes (NHC), and imidazolium ionic liquids.^74,323–327^ For example, cycloaminomethylation of indole with PFA gives access to 3-alkyl(phenyl)-3,4-dihydro-2*H*-1,5-(metheno)[1,3]benzodiaze-pines^328^ and quinazoline derivatives are accessible through condensation of anilines in the presence of HCHO, derived from PFA, in the presence of a Schiff-base catalyst.^96^ Macrocycles, such as diaza tetraacetylenes, can be synthesised from α,ω-diacetylenes with PFA as the HCHO source in the presence of copper chloride as a catalyst.^329^ Other studies include the catalytic *in situ* formation of formaldehyde from methanol for the synthesis of aminals,^330^ or benzimidazoles.^331^ In a different context annulations of carbo- and N-heterocycles with formaldehyde under metal-catalysed or metal-free conditions were also demonstrated.^332–334^

Last but not least, metal hydroxides of *i.e.* calcium or lead in the presence of trace amounts of carbohydrates form enediol metal complexes which then induce the Formose reaction in the presence of formaldehyde leading to the formation of mixtures of carbohydrates such as glycolaldehyde, tetrose, and glyceraldehydes.^7,15–20^

### Formaldehyde as a surrogate for carbonylation

Similar to the above mentioned reactions, FAH and PFA are also convenient alternatives to gaseous reagents (*i.e.* CO, syngas) for carbonylation reactions. In this context, these reagents serve as CO-surrogates that offer the possibility to perform reactions on the labscale without the specific infrastructure required for highly hazardous reagents based on carbon monoxide. Thus, for example hydroformylation, aryloxy/alkoxycarbonylation or *N*-formylation can be easily performed using such CO-surrogates to introduce functional groups like aldehydes, acids, esters or amides, enable the synthesis of heterocycles, introduce chiral centres or isotope-labelled entities (Table 3).^52,54,98,112,113,335–339^ An interesting study in the field of carbonylation reactions involving paraformaldehyde, showed that PFA can react with syngas (70 bar, 100 °C, 20 h) in the presence of a rhodium pincer complex and potassium formate forming ethylene glycol in moderate yields (40%).^340^

**Table 3:** Examples for carbonylation with PFA in the presence of water catalysed by molecular metal complexes

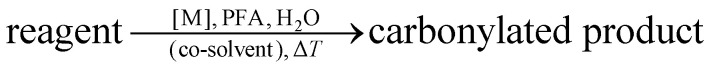						
Entry	Catalyst	*T* [°C]	Reagent	Product	[%]	Ref.
1	Rh	≤120	Alkene	Aldehyde^a^	≤96	110, 113, 114, 337 and 341–344
2	Pd	110	Bromo-biphenyl	Ketone	≤75	345
3	Ru or Pd	100–130	Alkene + alcohol	Ester^a^	≤93	105, 106 and 117
4	Rh	110	PhOH + PhI	Arylester	≤86	94
5	Cu, Ir or Ru	25–60, 115, 150	Amine	Amide	≤99	100, 112 and 115
6	Rh	100	Aniline + alkyne	Quinoline	≤95	346
7	Co + Ag	120	Aniline + ketone	Quinoline	≤91	347

a Typically with a high region-selectivity for terminal carbonylation of olefins.

### Hydrogen evolution and transfer hydrogenation reactions

Labscale synthesis often requires specific equipment and infrastructure to handle (hazardous) gases under safe conditions. In this regard, formaldehyde (6.7 wt% H_2_) and paraformaldehyde (per unit: 6.7 wt% H_2_) as liquid/solid organic hydrogen carrier (LOHC/SOHC) molecules are interesting as they can generate hydrogen on demand, *in situ*, with less sophisticated reactors and glassware. Nonetheless, gas sensors (leakage) and appropriate reaction vessels should be used for the corresponding reactions and the expected gas amount should be considered for the reaction setup and the reaction vessel.

Aq. formaldehyde (formaldehyde hydrate (FAH), methanediol; H_2_C(OH)_2_)) and paraformaldehyde (PFA) in water have been studied for hydrogen evolution and transfer-hydrogenation reactions. The high hydrogen content of methanediol (8.4 wt% H_2_) and the exothermic and exergonic decomposition to hydrogen and carbon dioxide enables fairly mild conditions (<100 °C) with molecular metal catalysts (Tables 1–4).^28,30,66–71,348,349^

**Table 4:** Examples for low-temperature hydrogen evolution from FAH and PFA catalysed by molecular metal complexes^28,30,66–71^

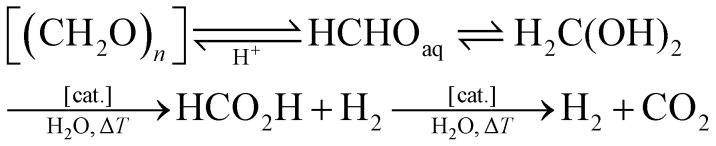						
Entry	Metal complex	*T* [°C]	pH	TON	TOF [h^−1^]	Ref.
1	Ru	95	5.5–7	700	3142	28 and 30
2	Ir	95	11	178	n.d.	69
3	Ir	60	11	51	n.d.	70
4	Ru	60	>12	1787	20 000	68
5	Ru	95	7	24 000	8300	71
6	Ru	95	n.d.	12 905	5715	66
7	Ru	90	n.d.	n.d.	685	67
8^a^	Ru	90	n.d.	n.d.	1650	350

a Immobilised on a covalent triazine framework (CTF).

These promising results let then to further evaluation of FAH and PFA in water for metal catalysed transfer-hydrogenation enabling the reduction of functional groups (*i.e.* C=C,^103^ C≡C,^61^ C≡N,^60^ C=O,^108^ excerpts in Table 5). Interestingly, the use of D_2_O or deuterated PFA gives access to the regioselectively isotope-labelled/deuterated products. Moreover, bi-catalytic systems were also developed for hydrogen evolution from methanol, wherein these approaches formaldehyde is generated *in situ* from initial enzymatic methanol activation in particular, and coupled with metal-catalysed hydrogen evolution (*i.e.* Ru, Ir) at low temperature.^228,229,348,351,352^ In these studies Ru-MOFs as catalysts were also successfully tested to achieve good catalyst-enzyme compatibility along with good stability in the presence of formaldehyde (H_2_ production: 106 mmol h^−1^ mol^−1^_Ru_; pH 7.5 at 35 °C).^351^ The latter is important, since enzymes can also undergo deactivation in the presence of formaldehyde.^353^

**Table 5:** Examples for transfer-hydrogenation with PFA in the presence of water catalysed by molecular metal complexes

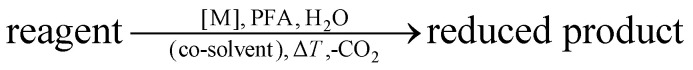						
Entry	Catalyst	*T* [°C]	Reagent	Product	[%]	Ref.
1	Ru	110	Enone	Ketone	≤93	103
2	Fe	120	Aldehyde	Alcohol	≤89	108
3	Ru	80	Alkyne	*E*-Alkene	≤93	61
4	Ru	90	Nitrile	Alcohol	≤93	60

### Formaldehyde in relation to biomass

In essence, biomass is all around us: everything that is living and everything that once lived or pertained to a living organism; from fallen tree leaves, to algae or domestic biowaste.^354^ With such abundance, it is no wonder that biomass has become a widely popular choice for energy production.^355^

**Scheme 21:**
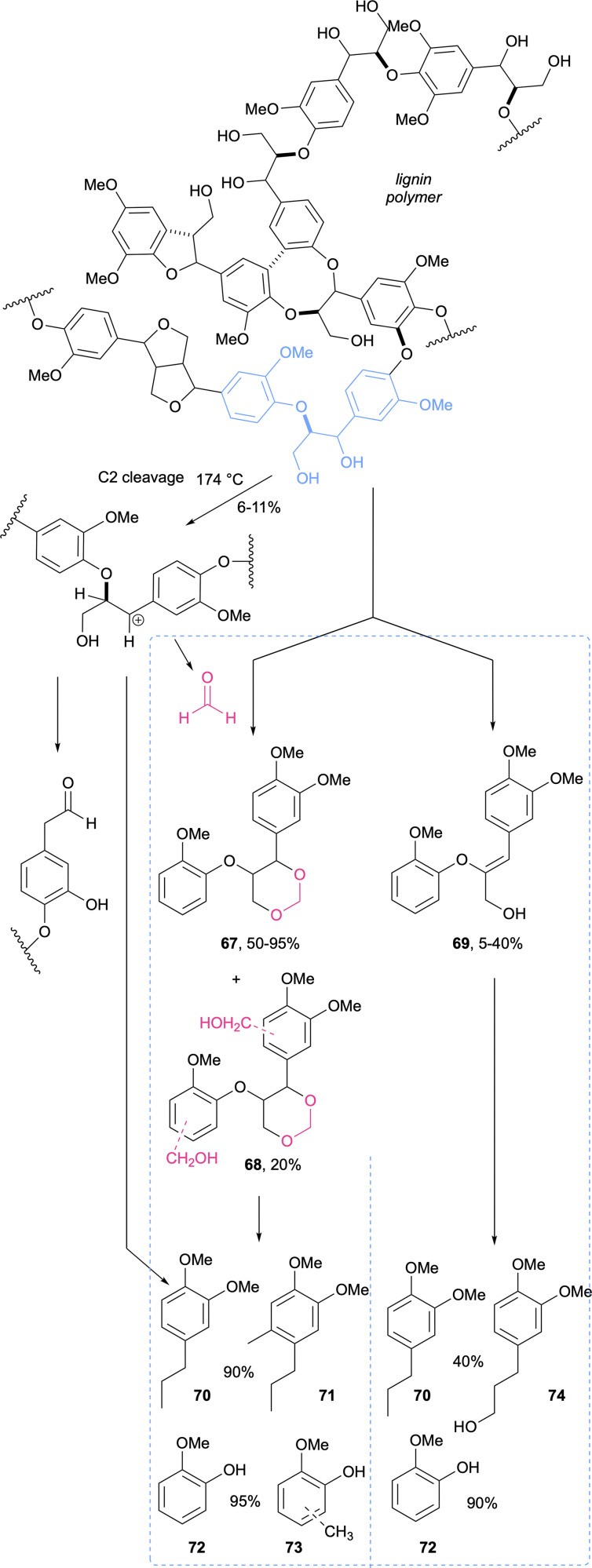
Lignin is one of the key biopolymers in woody biomass. Formaldehyde (pink) – which can also be generated in lignin acidolysis (left side route) – stabilizes the process of reactive lignin extraction and hydrogenolysis and provides for higher yields in monomer production (dotted square, left); compared to the lack of formaldehyde (dotted square, right). These studies used veratrylglycerol-*b*-guaiacyl (in blue in the lignin polymer) as a model compound.^360,361^

Chemically, biomass is primarily composed of carbohydrates (cellulose and hemicellulose) and the complex organic polymer lignin.^356,357^ Lignin, of all the biomass components, has the highest energy density and is a great source for the large-scale production of aromatic compounds.^358^ However, the huge potential of lignin has been stagnant for decades, given the lack of high-yielding depolymerisation methods.^359^ It is common that ether bonds in lignin cleave with acids or high temperatures, forming stable C–C bonds leading to “condensed lignin” and poor monomer yields (70–74). Formaldehyde allows for the stabilisation of lignin during extraction by hindering the formation of these stable C–C bonds, a process that can happen in two ways: by forming stable six-membered acetals (67) with lignin's 1,3-diols and by blocking electron-rich positions in the aromatic rings (Scheme 21).^360^ This development could be further exploited in industrial biomass processing, given that lignin-borne formaldehyde is generated during the thermal processing of wood,^160,361^ or through lignin acidolysis.^362^ To improve the depolymerisation of lignin it has been also demonstrated that the addition of ethanol as a formaldehyde scavenger is beneficial and as a by-product diethoxymethane is formed which is interesting as a biofuel.^363^ In the ever-growing sector of biomanufacturing, lignocellulose (and other sugar-based) feedstocks, which require tedious pre-treatments, are being shunned in favour of C1 compounds.^209^ Of these, methane, methanol, CO_*x*_ and formate have been at the lead of fossil fuel replacement.^364^ Formaldehyde was far from being included in this list, and in fact, for years, the only reports available would be on formaldehyde as a primary pollutant from biomass burning.^163,365^

Still, recent studies have showcased the potential of a symbiotic biomass exploitation. Thus, the catalytic oxidation of biomass-derived polyols, such as glycerol, constitutes a promising and sustainable alternative. Recent studies have showcased this possibility using different, high-yield strategies. In this way, formic acid and formaldehyde (15% over TiO_2_/O_2_;^366^ 21% over Co@TiO_2_)^367^ have been successfully obtained through photocatalytic degradation of glycerol. The major product of polyol/glycerol degradation in photo-, thermo- and electrocatalytic methods from biomass or their hydroxylated derivatives remains formic acid with high selectivity.^366–369^ While formic acid production from biomass remains the more attractive option, overoxidation to CO_2_ can significantly limit the yields.^370,371^ By coupling a second reaction that converts methanol into valuable formaldehyde, researchers have been able to significantly improve yields by twofold by producing two valuable end products: formic acid and formaldehyde.^371^ To this end, formaldehyde is starting to be known as a very valuable sub-product.^372^

All in all, formaldehyde remains a supporting actor in the biomass industry, with other C1 compounds in the main roles. Yet, realistically, most of these single-carbon substrates are naturally converted into formaldehyde in the first step of their assimilation process,^370,373^ a fact that places formaldehyde in the ideal position for the creation of a highly energy efficient, highly valorisable cycle.^374,375^ Notably, biomass-derived formaldehyde, from biomethanol oxidation, is economically viable according a LCA from 2024.^372^ For the valorisation of the biomass lignin, formaldehyde will continue to play an important role in particular for the production of renewable lignin-phenol-formaldehyde or lignin-urea-formaldehyde resins to complement and substitute fossil-based formaldehyde resins for example (Scheme 6).^376–394^

## Conclusion, perspectives and outlook: the role of formaldehyde in a defossilised society?

Formaldehyde and surrogates are under investigation in diverse fields of novel and established applications. Historically, this also covers the modernisation of conventional production methods such as fossil-based resins to lignin-based resins (UNSDG 9 and 12). Likewise, classical synthetic methods in organic chemistry are further explored along with new catalysts and biomass-derived substrates under thermo-, electro-, photo- and biochemical conditions. The formaldehyde industries together with the methanol sector have promising opportunities for defossilisation of the required feedstocks through CCUS and biomass valorisation. In this context, photocatalytic and electrocatalytic processes driven by renewable energies for the carbon dioxide reduction to formaldehyde and methanol are important keys to enable the defossilisation of these industrial branches. These production pathways will be likely complemented with more efficient and sustainable formaldehyde production processes with renewable methanol and CCUS-derived syngas as feedstocks. In the energy and transportation sectors renewable formaldehyde together with methanol are of interest owing to their relatively high energy and hydrogen content which is of relevance not only for both stationary and mobile energy storage for fuel cells, but also for synthetic fuels such as renewable oxymethylene ethers for combustion engines (UNSDG 7 and 13).

The successful implementation of technologies for the production of renewable formaldehyde and methanol along with the substitution of fossil reagents by biomass derived reagents enables the opportunity to use existing chemical production infrastructures with defossilised C1-feedstocks (UNSDG 9). Furthermore, new technologies for formaldehyde degradation are of interest to improve the purity of waste streams, water, soil and air through formaldehyde detoxification (UNSDG 3, 6, 13–15).

For further advancement of formaldehyde-based production and utilisation technologies, the above discussed methodologies under development will require more techno-economic analysis (TEA) and life-cycle assessment (LCA) to enable higher technology readiness levels (TRL) and industrial applications. To date, TEA and LCA are very limited for new technologies with formaldehyde as a key molecule for *i.e.* DME/OME fuel production, renewable HCHO from biomass or HCHO degradation,^372,395,396^ TEA and LCA with a focus on HCHO for hydrogen storage and within the CCUS sector are still required for further advancement. In general, LCA and TEA can be powerful tools to determine whether a specific element, process or area is functioning satisfactorily or is beneficial for the producer and end-user. While this review does not address the economy from a monetary perspective, we offer chemical insights into the benefits of using formaldehyde and its surrogates as a C1 platform for a defossilised society. For the exploration and discovery of new methods for formaldehyde formation and utilisation one needs to consider also the limitations of the reaction conditions (*i.e.* temperature, pH sensitivity and water content) as summarised in this tutorial.

All these measures and advancements for the defossilisation of the chemical industries over the next decades will require and will lead to more innovation and simultaneously improve the level of education and knowledge (UNSDG 4). In this way, we can conclude that the potential of formaldehyde is high on different counts. First and foremost, as a defossilising agent, by greatly improving our society through its surroundings; but also by promoting innovation through sustainability, given that a great potential of formaldehyde comes from natural, recyclable and reused sources. For all these reasons, we consider that renewable formaldehyde is an excellent asset to develop greener, richer and more sustainable societies.

## Author contributions

Concept: MHGP; writing – original draft: MHGP; writing, review & editing: MHGP, JD and AR.

## Conflicts of interest

A patent has been filed under 10 2013 011 379.2 at the German Patent Office DMPA (WO2015/003680A003681; PCT/DE002014/000344).

## Data Availability

All data included in this article are based on original data which are available through the cited literature in the references.
